# Satellite Data Potentialities in Solid Waste Landfill Monitoring: Review and Case Studies

**DOI:** 10.3390/s23083917

**Published:** 2023-04-12

**Authors:** Lorenzo Giuliano Papale, Giorgia Guerrisi, Davide De Santis, Giovanni Schiavon, Fabio Del Frate

**Affiliations:** 1 Department of Civil Engineering and Computer Science Engineering, Tor Vergata University of Rome, 00133 Rome, Italy; 2 GEO-K s.r.l., 00133 Rome, Italy

**Keywords:** remote sensors, satellite landfill monitoring, waste disposal, waste management

## Abstract

Remote sensing can represent an important instrument for monitoring landfills and their evolution over time. In general, remote sensing can offer a global and rapid view of the Earth’s surface. Thanks to a wide variety of heterogeneous sensors, it can provide high-level information, making it a useful technology for many applications. The main purpose of this paper is to provide a review of relevant methods based on remote sensing for landfill identification and monitoring. The methods found in the literature make use of measurements acquired from both multi-spectral and radar sensors and exploit vegetation indexes, land surface temperature, and backscatter information, either separately or in combination. Moreover, additional information can be provided by atmospheric sounders able to detect gas emissions (e.g., methane) and hyperspectral sensors. In order to provide a comprehensive overview of the full potential of Earth observation data for landfill monitoring, this article also provides applications of the main procedures presented to selected test sites. These applications highlight the potentialities of satellite-borne sensors for improving the detection and delimitation of landfills and enhancing the evaluation of waste disposal effects on environmental health. The results revealed that a single-sensor-based analysis can provide significant information on the landfill evolution. However, a data fusion approach that incorporates data acquired from heterogeneous sensors, including visible/near infrared, thermal infrared, and synthetic aperture radar (SAR), can result in a more effective instrument to fully support the monitoring of landfills and their effect on the surrounding area. In particular, the results show that a synergistic use of multispectral indexes, land surface temperature, and the backscatter coefficient retrieved from SAR sensors can improve the sensitivity to changes in the spatial geometry of the considered site.

## 1. Introduction and Literature Review

Over the last decades, the ever-growing world population and industrial production, human activities, and urbanization have led to an increase in waste production [1]. Improper waste management has a negative impact on human health and the environment. In particular, poor waste management practices can lead to serious contamination of the soil, water, and atmosphere [2,3]. Primarily due to economic advantages, landfills and/or open dump sites are still a common method for the disposal of municipal solid waste (MSW). In certain countries, such as those in the European Union, specific targets exist that aim to decrease the volume of waste sent to landfills in favor of more sustainable disposal methods [4]. However, in many developing countries, waste management is often a low-priority issue, and improper and unsustainable disposal practices are used. As a result, landfills are often not engineered, and the waste is indiscriminately disposed of in an unsanitary manner [5]. The potential impacts of landfills on the environment comprise soil, water, and air pollution, which mean impacts on human health, agriculture, and drinking water. Leachate is indeed one of the products of landfills. Leachate is caused by the infiltration of precipitations through the waste deposited on the ground, and it leads to the contamination of soil and groundwater. Additionally, gases produced by the decomposition of biological organic matter are released and contaminate the atmosphere if they are not properly collected. Landfill fires also contribute to the sources of pollution and can significantly harm the environment due to the emission of toxins into the atmosphere, soil, and water [4]. All these aspects are considered and monitored in a regular engineered landfill; however, this does not happen with an illegal dumping site, and the consequences for humans and environment can be disastrous. In this context, landfill monitoring represents a crucial activity, but in situ investigations are often time-consuming and require high costs. Indeed, they are often characterized by several difficulties such as access to the sites, the transportation and positioning of the instruments, analyses, and time scales [6]. Furthermore, landfill monitoring becomes even more complex for illegal dump sites, which usually arise at unknown sites and within a short timeframe [7].

Remote sensing (RS) can provide feasible and economic solutions for the acquisition of valuable data for the detection and monitoring of dumps and landfill sites and to assess their effect on the environment. For this purpose, various RS satellite sensors and data can be utilized together with different analysis techniques. The earliest works introducing spatial technologies for landfill monitoring adopted geographic information systems (GISs) for solid waste management by combining multi-source data, including RS acquisitions. Notably, RS data progressively changed this type of environmental monitoring procedure, first with aerial surveys photos and then with satellite acquisitions or by combining both data sources.

In the past, MSW monitoring was conducted by employing aerial acquisitions to perform visual inspections (e.g., [8]). Concurrently, satellite data have been adopted for the same purposes due to their data coverage. Moreover, despite the higher spatial resolution achievable with aerial acquisitions, satellite data avoids the surveys’ set-up and logistic costs and makes recurrent acquisitions over the same area available.

In 1973, a study [9] highlighted the potential of satellite RS, specifically the Earth Resources Technology Satellite (ERTS-1), with a multi-spectral scanner sensor on board, for monitoring large scale events such as dumping. In this particular case, the authors used spacecraft data to observe a large ocean dump area adjacent to the New York Metropolitan area. A subsequent work [10] also demonstrated the utility of GIS and RS data for the identification of suitable sites for the storage of industrial waste. In the procedure suggested for performing the selection, they considered the use of aerial photography and the Landsat multi-spectral scanner and thematic mapper to obtain land cover and land use information. Additionally, the approach of combining aerial survey acquisitions and optical images collected by satellites was adopted by Oluić et al. [11] by performing a structural–geologic analysis of an MSW site through a visual inspection of Landsat ETM/aerial imagery and geophysical data integration. Furthermore, in 2009, Biotto et al. [12] employed Ikonos data to correct and update the infrastructure plan obtained from the official regional map in order to detect illegal landfills. In 2015, Lucendo-Monedero et al. [13] developed a model for the detection of areas with illegal landfills using logistic regression. Notably, a set of potential areas with illegal waste disposals in Andalusia were analyzed using logistic regression to predict the occurrence of these areas by jointly involving geographical and behavioral variables. In particular, the uncontrolled landfills used for this study were chosen from sites previously detected by RS and aerial photography. 

Several methods for dump detection and analysis involve images acquired by means of multispectral sensors onboard satellites. As discussed below, multispectral images can be analyzed directly by studying the spectral signature trends or used to retrieve multispectral indexes, and they are often integrated with GIS or ground-based information. Authors have pointed out [14] three main aspects utilized to sense dumps from RS images: thermal anomalies, injurious effects on vegetation, and spectral signatures. 

**Figure 1:**
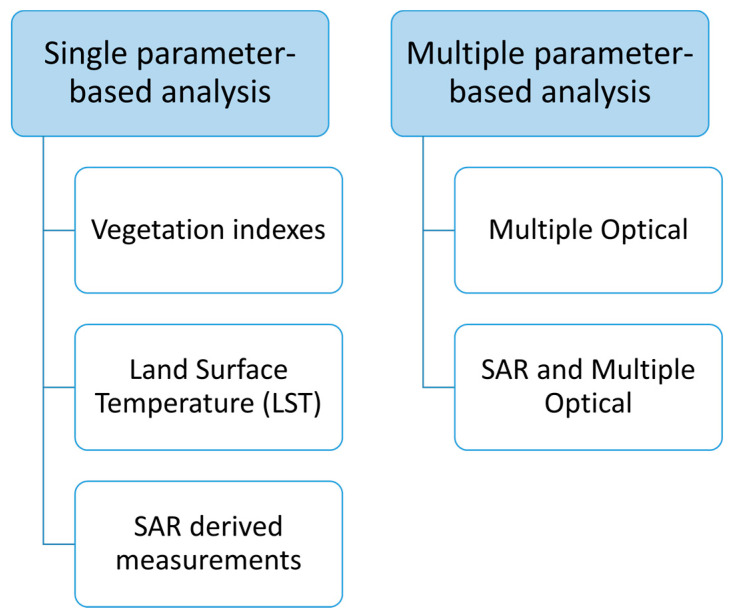
Summary diagram of the methodology for presenting the review results. Revised manuscripts are categorized into two main blocks: the “single-parameter-based analysis” and the “multiple-parameter-based analysis”. Each block consists of different sub-classifications.

The aim of this article is to review and collect the most significant works on the identification and monitoring of waste dumping sites using satellite RS technologies. Section 1 introduces the review and presents its outcomes, which are classified according to the measurement typology. In particular, the research works are categorized according to the classification reported in Figure 1. This includes two main classes: the single-parameter-based analysis and the multiple-parameter-based analysis. The former, described in Section 1.1, includes studies that distinctly used information from a specific portion of the electromagnetic spectrum, while the latter includes studies that combined such information. These and are described in Section 1.2. The single-parameter-based analysis includes the following sub-classes:

- Vegetation-indexes-based analysis (presented in Section 1.1.1): works that mainly use vegetation indexes for the identification and monitoring of the landfill sites are discussed in this section. In more detail, the considered indexes include the normalized difference vegetation index (NDVI), normalized difference water index (NDWI), and other specific indexes that can be obtained through multispectral sensors.
- Land-surface-temperature-based analysis (presented in Section 1.1.2): this section reviews the papers that use land surface temperature (LST) for analyses related to a landfill site. LST is derived from thermal infrared channel of the sensors on board the Landsat satellites.
- SAR-based analysis (presented in Section 1.1.3): this section includes the papers that use synthetic aperture radar (SAR) data.

The multiple parameter-based analysis, instead, includes the following sub-classes:

- Multiple-optical-parameters-based analysis (presented in Section 1.2.1): works that use the LST in conjunction with vegetation indexes are included in this section.
- SAR- and Multiple-optical-Parameters-based analysis (presented in Section 1.2.2): this section includes the papers that use SAR data combined with multispectral optical sensors and vegetation indexes or LST measurements.

Thereafter, Section 2 presents the application case studies. We applied the most common state-of-the-art techniques to known landfill sites in order to test the capabilities of the specific procedures and to provide a comprehensive overview of attainable results from sensor-based satellite measurements. This section includes a description of the sensor-based indexes used for the analysis and the obtained results. Finally, Section 3 provides the conclusion.

It must be noted that in this review, we focused on the use of satellite RS data that represent the most widely applied category of data due to their wide distribution, availability, global coverage, and ease of access. Nevertheless, a few methods based on aerial platforms also exist [15], and monitoring by means of unmanned aerial vehicles (UAVs) is increasingly being adopted [16,17]. It is also worth mentioning that RS of the atmosphere can also represent a valid instrument for landfill studies by exploiting the gas emissions from dumping sites. Thus far, methane (CH_4_) emissions were used by [18] to locate landfills using data acquired by an aircraft.

### 1.1. Single-Parameter-Based Analysis

This section describes the works that separately exploit satellite-sensor-derived information acquired at the visible–near infrared–short wave infrared (VIS-NIR-SWIR), thermal infrared (TIR), and microwave parts of the electromagnetic spectrum, respectively. It consists of the following subsections: the vegetation-indexes-based analysis, land-surface-temperature-based analysis, and SAR-based analysis.

#### 1.1.1. Vegetation-Indexes-Based Analysis

A method was proposed by [19] for landfill monitoring by exploiting both satellite and ground-based data. Multispectral satellite data acquired by means of very high resolutions (between approximately 2 and 0.5 m), Pleiades and WorldView2 sensors, and aerial orthophotos were used to identify points of interest for monitoring the area. Two spectral indexes were analyzed: the NDVI and the global environmental monitoring index (GEMI), which equally depends on reflectance in the visible–near infrared range but is less affected by atmospheric effects and the type of sensor used, although it is more sensitive to bare soil variation than the NDVI. These indexes were used for a multi-temporal change detection analysis of the area. The obtained information was applied to enhance in situ geomatic surveys. Results obtained for a study site in Calabria, Italy, demonstrated that satellite data can effectively support land surveys, detecting critical points and highlighting most important changes.

Additionally, Ref. [20] utilized the relationship between dump-induced contamination and vegetation stress to identify uncontrolled landfills and validated their technique on the watershed area of the Venice lagoon, Italy. They integrated GIS and RS information acquired by the multispectral, pan-sharpened IKONOS sensor with a spatial resolution of 1 m. RS imagery was used to identify stressed vegetated areas associated with the dump. Specifically, information available from local authorities was used to identify known illegal landfills and as training and calibration information for a machine learning algorithm for the classification of stressed vegetated areas. The automatic classification method was integrated with a visual interpretation of aerial photographs and GIS information, such as road networks and population density. As a result, vegetation stress proved to be a good indicator for illegal landfill detection, and RS proved to be a successful technique for landfill identification.

Vegetation stress induced by the presence of dump sites is also an element of study in [21], in which several multispectral vegetation indexes were compared to identify the most suitable index for assessing environmental risks caused by MSW open dumps. The authors analyzed NDVI, SAVI, and MSAVI (modified soil-adjusted vegetation index, a modification of SAVI) obtained from Landsat 8 acquisitions over dump areas in Pakistan and compared them with three different criteria. The indexes’ variation was studied in comparison to the distance from the source of contamination, finding that before the start of the dumping, no variation in vegetation health was observed in the dumps’ surroundings. On the contrary, after MSW dumping, it was found that vegetation stress increased as the distance from the landfill decreased. They found that the MSAVI is the most suitable index for this purpose.

The combination of GIS and RS is also presented in [22] and for risk assessment. The former developed an algorithmic criterion to compare the hazardous effects induced by different MSWs and utilized GIS to organize input data and very high-resolution data from QuickBird to obtain land cover maps of around the study area. The latter generated a risk assessment map of illegal waste-dumping areas by combining GIS and RS information. They used multispectral images acquired by the FORMOSAT-2 satellite to analyze the spectral characteristics of dumping areas, known from field surveys and EPA (Environmental Protection Administration) archives. They integrated this information in a risk assessment model composed of other geospatial factors related to waste dumping. The integration of spectral features greatly improves the risk assessment mapping results, although higher spectral resolution imagery may be useful in avoiding the misclassification of waste materials with bare soil.

Additionally, Ref. [14] applied spectral features to investigate the possibility of identifying landfill areas using Landsat 5 satellite images, digital orthophotos, and a Corine 1990 land cover map. They analyzed the spectral signature in all seven bands of the thematic mapper sensor for eight classes of interest (including the “dump” class) individuated using the ISODATA (iterative self-organizing data analysis) automatic pre-classification technique. A multitemporal comparison of the images allowed the authors to discriminate the dump spectral signature from the others, including those related to classes with similar behavior, such as urban area class. The digital orthophotos and land cover map were used as an aid for the elaboration, validation, and interpretation of the Landsat 5 data.

A change detection procedure was developed by [23] that aimed to distinguish waste burial sites. Two acquisitions from Landsat 7 and Landsat 8 were processed by means of the GRASS (geographic resources analysis support system) GIS, according to two methodologies. Similar to [14], the first procedure made use of an unsupervised classification method to group pixels with a similar spectral signature and then classified them with a maximum likelihood classifier. The second method was based on the tasseled cap transformation and discriminated the most changed areas by means of a “Greenness empirically normalization index” obtained from the greenness vegetation index. To validate the method, ground truth maps were realized through a comparison and visual analysis of available archive images with a high spatial resolution (from 1 m to 0.18 m). This second method led to significant and applicable results.

A method to detect micro-dumps and greenhouse gases through the use of satellite RS data was developed by [24]. In particular, the suggested procedure was applied to Pleiades multispectral data with a spatial resolution of 2 m in the RGB and near infrared bands. The authors exploited both the spatial and spectral features extracted from the images through the scattering transform algorithm. The spatial characterization was achieved by extracting textural features from the images, while the spectral features were based on the brightness information. The obtained results showed good performance in greenhouse gas detection; on the contrary, the results of the micro-dump recognition, characterized by a small dimension and spectral and spatial inhomogeneity, reported a high false alarm rate.

Finally, Ref. [25] proposed a method for identifying and ranking landfill sites using the city of Faisalabad as a case study. Indeed, the city is one of the largest industrial centers in Pakistan, and solid waste derived from its huge production is often dumped randomly near populated areas. RS data and GIS were used to rank the areas for developing or expanding an existing landfill site from the most suitable sites to the worst sites. As ranking criteria, three indexes derived from Landsat 8 acquisitions were used in addition to physical factors, including water bodies, roads, and the population. The indexes were the NDVI, NDWI, and the normalized difference built-up index (NDBI). The latter was used to assess the urban built-up size, the geographical distribution of the study area, and a comprehensive picture of the urban land cover. Additionally, Ref. [26] utilized the NDVI and land use (LU)/land cover (LC) maps for the identification and ranking of possible dumping sites in Saudi Arabia, using Sentinel-2 data. The obtained maps and NDVI were integrated with other geophysical and spatial information to obtain a set of information for selecting the most appropriate site for waste disposal.

#### 1.1.2. Land-Surface-Temperature-Based Analysis

The effectiveness of a multitemporal analysis of the LST, obtained by Landsat satellites monitoring landfill sites, was proven by [27]. In this work, the study area, data, and methodology proposed were used with the aim of determining the waste-dumping regions in a landfill using Landsat Thematic Mapper and Enhanced Thematic Mapper Plus images. Images that covered a temporal range of 10 years were collected during the summer and winter seasons. The results demonstrated the effectiveness of the LST for determining waste location, especially considering the summer acquisition when the heat flux is more pronounced.

Landsat data were also used in [28]: they successfully developed a methodology for identifying landfill fires by correlating them with landfill temperature anomalies. From Landsat 5 and Landsat 8 images, they obtained LST maps over a 17-year period. Also, Ref. [29] estimated LST using Landsat data acquired from 2000 to 2016 to demonstrate the correlation between this parameter and the amount of solid waste buried in the landfill. Subsequently, they used landfill-site-specific information in addition to other meteorological parameters to estimate the LST with a neural network algorithm.

In [30], the authors assessed the LST distribution for two large landfill sites in Vietnam using Landsat 8 satellite images. They derived the LST from two images, acquired in 2019 and 2020, that showed the two different test sites. They proved that the landfill surface temperature is often higher than 40 °C and is much higher than the surrounding area, even compared to built-up areas. Additionally, the results show that the difference between the highest LST (at the landfill) and the lowest LST (at the area covered by vegetation) in both test areas was about 20 degrees.

#### 1.1.3. SAR-Based Analysis

Ali et al. [31] used a single Radarsat-1 C-band SAR acquisition to monitor multiple MSW sites in the city of Riyadh, Saudi Arabia. In particular, they processed one Radarsat-1 backscattering image (in F2 mode), and a digital elevation model (DEM) was obtained using stereo-SAR techniques to orthorectify the image. Eventually, image segmentation was performed to map waste disposals by applying a supervised classification algorithm in PCI software version 10, using ground-truth samples as input. The results confirmed that satellite SAR imagery and digital image processing techniques could be valuable for MSW site monitoring.

This study used the SAR backscatter to monitor waste disposal areas; the employment of SAR interferometry for the same purpose is also found in literature. Indeed, since several factors related to the observed surface affect the SAR backscatter signal, the combination of multiple images can provide more robust information on the characteristics of a landfill. For example, Ref. [32] monitored the change in waste disposal volume in a landfill located in Athens by using two pairs of ENVISAT ASAR images and applied the SAR interferometry technique to generate two DEMs. The two DEMs, referring to the first and (assumed as reference product) second dates, respectively, were subtracted to derive variations of the elevations. In particular, changes in the waste volume were examined using elevation profiles obtained with the two DEMs.

The interferometric SAR (InSAR) technique was also applied in [33] in combination with unmanned aerial vehicle (UAV) photogrammetry and ground measurement in order to obtain information on landfill deformation and evaluate the risks related to the waste disposal site. The authors considered one of the largest engineered sanitary landfills in China, the Tianziling landfill, as a study case. In particular, Sentinel-1 data were used to assess deformation during the monitoring period, supported and integrated with ground-based and UAV data. The multi-source information was then used to identify the potential subsidence area of the landfill for the risk assessment analysis. The results showed that the approach permitted the detection of deformation in the range of millimeters to meters, allowing for the identification of unstable areas for risk estimation. In [34], the authors used a differential InSAR technique for monitoring landfill settlements in the Montegrosso-Pallareta landfill in Italy. They used multi-temporal COSMO-SkyMed interferometric data to monitor a low-magnitude landfill settlement with millimetric accuracy. In [35], the authors used the multitemporal InSAR technique for the time-series monitoring of deformation at the Xingfeng landfill in China. Multitemporal InSAR allows for the elimination of the influences of temporal and spatial decorrelation and atmospheric noise. The authors used images from Sentinel-1A, Radarsat-2, ALOS-2, and TerraSAR-X/TanDEM-X to measure the settlement and thickness of the landfill. The results showed that the coherence of the landfill during the initial stage of closure is primarily affected by temporal decorrelation, caused by considerable gradient deformation rather than by spatial decorrelation. Moreover, the results revealed the correlation between the time of settlement and thickness, with the settlement of younger areas usually found to be greater than that of older areas.

### 1.2. Multiple-Parameter-Based Analysis

This section describes works that combine satellite-sensor-derived information acquired at the VIS-NIR-SWIR, TIR, and microwave parts of the electromagnetic spectrum, respectively. It consists of the following subsections: multiple-optical-parameters based analysis and the combination of SAR and multiple-optical-parameters based analysis.

#### 1.2.1. Multiple-Optical-Parameters-Based Analysis

Several authors proposed an approach that makes use of both temperature information and multispectral indexes. The methodology proposed in [36] follows the method already examined in [27] except for the use of different study sites and the introduction of an NDVI visual analysis. From Landsat Thematic Mapper data, they derived LST and NDVI images for a total of 81 acquisitions in different seasons throughout the year. The results confirmed that the LST of the landfill is higher than the surrounding vegetation and air temperature, and that this difference is more pronounced during the spring and summer seasons. In particular, they found that the LST is generally higher for an active landfill compared to a closed one, and it is higher for open landfill stages than capped landfill stages. Nevertheless, the LST was found to be affected by moisture caused by rain or snow. In fact, precipitation provides a suitable environment for bacteria to proliferate, inducing a high rate of gas production and heat generation. Finally, the NDVI was used to analyze vegetation conditions in and around the landfill.

Additionally, Ref. [21], following their previous work [21], integrated the LST into the analysis of vegetation indexes with the aim of evaluating the bio-thermal influence of two dumping sites in Pakistan on the surrounding areas. The results previously obtained for the vegetation indexes were confirmed; moreover, they used Landsat 8 data to demonstrate that the LST decreases moving away from the sites.

In 2008, Yang et al. [37] combined RS data in a GIS environment to analyze the leachate and gas emissions from landfills in a metropolitan area of Jiangsu Province, China. Particularly, the LST, the NDVI, and the CMI (clay minerals index) were considered in the study. According to the authors, these choices were motivated by the fact that differences in the LST between actual landfill sites and the surrounding areas are correlated with the speed and significance of the biodegradation processes occurring in the landfill and the release of the related gases. The NDVI, due to its correlation with the water content in the soil, acts as an indicator of the presence of leachates; the CMI allows for an assessment of the clay concentration in the surface since this material is commonly used to prevent the release of contaminated water and other liquids by confining landfills. Concerning the employment of RS data, a Landsat-7 Enhanced Thematic Mapper Plus (ETM+) image was retrieved and processed to compute the LST and the multispectral indexes. Eventually, the obtained results were combined with available digital maps and regional data to perform a spatial analysis of the landfill areas by examining their environmental conditions and distance from water bodies and infrastructures.

In 2018, Sarp et al. [38] analyzed the impact of an abandoned aggregate quarry used for uncontrolled waste disposal on groundwater, the LST, land surface moisture, and vegetation cover using GIS and RS technologies. In particular, for each of these domains, an index derived from the Landsat 8 imagery was selected for the years 2014 and 2017. In detail, the Landsat 8 sensor TIRS was employed to compute the LST and assess the changes that occurred during the specified time interval as an indicator of the influence of solid waste deposition on thermal absorption and emissions. Moreover, the multispectral indexes NDMI and SAVI were used to assess the impact of solid waste disposal on surface moisture and surrounding vegetation, respectively. Furthermore, Pearson’s correlation coefficient was utilized to evaluate the degree of correlation between the variables mentioned above, i.e., LST, NDMI and SAVI, for both the time periods: 2014 and 2017. Eventually, groundwater contamination was assessed in the study area, with samples taken from four wells close to the site. According to the obtained results, solid waste disposal caused groundwater pollution, increased surface temperature, and reduced soil moisture.

In 2021, Ganci et al. [39] proposed the employment of multispectral satellite images to characterize the state of activity of landfills since, according to the authors, when active, waste disposal sites show differences in surface conditions compared to neighboring areas. Indeed, regarding the usage of RS data, Landsat (4,5,7,8), EOS-Aster, Sentinel-2, and Doves-Planetscope acquisitions were used to derive the LST, NDVI and NDWI as indicators of the surface temperature, state of vegetation, and soil moisture, respectively. In particular, the employment of the NDWI allowed for an analysis of the soil moisture to identify the leachate contamination of the aquifers. Preliminary results, obtained by combining the indexes computed for the site and the surrounding areas, show how this approach can identify known the areas and levels of activity of known landfill sites.

The LST was also used in combination with other parameters in [40]. In this work, the authors presented a characterization of the largest uncontrolled dump site in Egypt. They used RS data and other geological and geophysical data. They used Landsat 5 and Landsat 8 images to derive the LST, soil moisture maps, and LU/LCmaps. The LU/LC maps were used to derive the population settlement increment and to correlate the LST and soil moisture maps with the propagation of the municipal waste dump sites. The soil moisture map was used to characterize the leachate flow, while the LST was used to manifest the dump site surface temperature and to correlate the temperature to the quantity of waste.

In the region of Saskatoon, Canada, Ref. [41] used satellite imagery and vector data to produce a probability map of illegal dump sites. The authors used the night-time light data from the visible infrared imaging radiometer suite (VIIRS) sensor, which is on board the joint NASA/NOAA Suomi National Polar orbiting Partnership (Suomi NPP) satellite, and the data obtained from the Landsat 8 satellite. The former were used to identify low-light regions with lower anthropogenic activities to identify suitable sites for landfill expansion, and the latter were used to derive the LST and the MSAVI index. Satellite data were combined with vector data that included highways, railways, and the locations of regular disposal sites to produce a probability map by means of the simple additive weighting (SAW) method. Related to the Landsat 8 satellite data, they found that the MSAVI is a significant factor for the detection of illegal dump sites, while the LST is less effective for the identification of smaller-scale landfills. Similarly, in [42], the authors considered the same study area to detect illegal dump sites using vector parameters and satellite images. The vector parameters were active, regular landfills and highways. Additionally, they used the LST, the enhanced vegetation index (EVI), derived from MODIS data, and the formaldehyde total column (HCHO), derived from the Aura ozone monitoring instrument (OMI). The EVI is an alternative to the NDVI that minimizes errors due to atmospheric conditions and canopy background noise. The results showed that the LST and highways are important parameters for the identification of illegal dump sites; the latter are important because road network intensity and accessibility appear to be important for their occurrence. The EVI is mined by the predominating uniform land cover of grassland and shrubs. Instead, the HCHO is instead less spatially sensitive, limiting its potential as an indicator.

#### 1.2.2. SAR- and Multi-Optical-Parameters-Based Analysis

Ottavianelli et al. [43] introduced a visual assessment of hyperspectral and SAR RS for solid waste landfill management in the UK. In detail, the ERS and Envisat SAR images were processed to investigate the backscatter and interferometric coherence sensitivity to structural changes due to a landfilling operation. Moreover, a hyperspectral scene acquired by the PROBA-1 main instrument, the Compact High-Resolution Imaging Spectrometer (CHRIS), was employed (as a red–green–blue composite) to monitor the activities carried out in a landfill. According to the authors, this proved to be more adequate than multispectral data.

Yonezawa [44] proposed the joint use of multispectral and SAR data by employing ALOS PRISM, AVNIR-2, and PALSAR acquisitions for monitoring waste disposal sites. In particular, through a visual inspection of pan-sharpened PRISM and AVNIR-2 images, junkyards were identified in the target area. Moreover, PALSAR backscatter images were also examined in HH polarization for both flight directions (ascending and descending) to identify surface changes at waste disposal sites by visual interpretation. Eventually, panchromatic and multispectral QuickBird data allowed for the identification of a waste-tire disposal, leveraging its difference in the spectral signature with the surrounding land cover types.

Cadau et al. [45] proposed an integrated system for landfill detection and monitoring using EO data, particularly by involving multispectral and SAR data. While optical data are used mainly for 2D monitoring purposes, SAR information is useful for detecting both 2D and 3D landfill variations. For this purpose, concerning optical data (RAPIDEYE and SPOT-5), the DDI index was introduced and tested, combining optical-derived vegetation indexes with image textural features. Furthermore, regarding SAR data, COSMO-Skymed interferometric pairs were processed. In particular, the HH and VV interferometric pairs (ascending and descending paths) were analyzed to extract the 3D surface elevation model. The authors stated that this procedure could be applied to structures characterized by a regular geometry (with high coherence values) such that the signal phase information allows for the detection of height variations. Additionally, Cadau et al. proved that, in general, the decrease in the coherence between different SAR image pairs is correlated to a change in the landfill geometry’s scattering mechanisms. Moreover, night-time ASTER images, particularly TIR bands, were processed to retrieve the LST trends and detect potential thermal anomalies. Furthermore, the study analyzed the presence of new potential landfills by analyzing the DDI map and ASTER LST map.

Moreover, Agapiou et al. [46] investigated the sensitivity of multispectral, hyperspectral, and SAR data for olive mill waste disposal monitoring. Concerning multispectral acquisitions, very high resolution (VHR) data were employed, including Pleiades (0.50 m), SPOT 6 (1.5 m), QuickBird (0.60 m), WorldView-2 (0.40 m), and GeoEye 1 (0.40 m) images. Particularly, the VHR images were processed by computing two indexes, combining the bands in the visible and VNIR part of the spectrum: NDVI and OOMW (olive oil mill waster index). Moreover, a PCA (principal component analysis) was tested to identify the optimum linear combinations of the original bands. Then, an IHS (intensity–hue–saturation) transformation was applied to perform the resulting image fusion. Eventually, the ISODATA algorithm and the LSU technique were used to discriminate the olive mill waste disposal by performing an unsupervised classification. A COSMO-SkyMed image (2.5 m) was processed to identify the disposal areas, which were distinguished as black targets due to low backscattering of the signal (a significant presence of water). Moreover, a hyperspectral EO-ALI image (30 m) was employed through fusion techniques with the SAR image. However, the olive mill waste disposal areas were not successfully distinguished despite the improved spatial resolution.

### 1.3. Summary and Discussion of the Literature Review

In the first section, we reviewed the role of satellite RS in landfill monitoring. The analyzed studies reveal that satellite data offer promising results and represent a relevant instrument for the aforementioned task. Satellite acquisitions deliver a global view of the area of interest at a low cost and within a short time frame, and different kind of sensors on board provide advanced information in various portions of the electromagnetic spectrum. The reviewed works were classified according to the information retrieved from the satellite acquisitions, and four main categories of analysis were identified: vegetation-indexes-based analysis, land-surface-temperature-based analysis, multiple-optical-parameters-based analysis, and SAR- and multiple-optical-parameters-based analysis.

The review revealed that satellite data are mainly used with the purpose of identifying and monitoring landfill sites, both regular and illegal ones, and to also estimate the hazardous effects of dumping sites on their surrounding areas and to assess their environmental impact. Only a few studies used satellite data for more specific analyses, such as monitoring landfill emissions, i.e., leachates and gases, estimating the amount of buried waste, identifying landfill fires, and monitoring waste from olive mills. This suggests that satellite data are mainly applied for the investigation of large-scale events instead of more refined analyses. In fact, the sensors used in the analyzed literature are mostly high-spatial-resolution sensors, e.g., those on board the Landsat missions. Fewer studies made use of the very-high-resolution instruments carried by the Pléiades, WorldView-2, SPOT 6, QuickBird, GeoEye 1, and COSMO-SkyMed satellites. This lower number of research studies may be due to the limited spectral resolution and data distribution policy. Most of the analyzed works exploited the information obtained from optical instruments for their specific purposes, while a smaller part used SAR acquisitions. Related to the former, the measurements and indexes calculated were almost always combined in order to take advantage of the heterogeneity of the information. The most common parameters used for the analysis were the NDVI, NDWI, and LST, which have proven to be effective in the identification and characterization of landfill areas. In relation to the NDVI and NDWI, the works also demonstrated the ability of the indexes to assess the negative effects of landfills on the vegetation of surrounding areas. Furthermore, the LST was also shown to be able to identify a dump site, and the correlation between an increase in this value and the presence of a landfill was demonstrated. A lower number of articles used indexes such as the GEMI, SAVI, MSAVI, NDBI, or CMI, which are very specific indexes mainly used to obtain secondary information for particular tasks. For example, the NDBI was used to discriminate buildings to define an urban area, and CMI was used to detect clay on top of a landfill. The DDI receives a separate mention, as it is not so common but was specifically developed for landfill identification. Multispectral images were also often used to locate landfills by deriving their spectral signature. However, this was found to be very similar to the case of built-up areas, and additional analyses were required for a correct discrimination, e.g., a multitemporal analysis. Concerning SAR acquisitions, they were often used in combination with optical images. SAR was used mainly for landfill monitoring purposes, exploiting the radar backscatter to observe variations in the surface scattering mechanism. Moreover, the interferometry technique was used to assess changes in the volume of the site and to derive DEMs of the area of interest. The optical images were used to facilitate the visual inspection and to obtain DDI or LST measures.

In general, the analyzed works provided successful results, especially those that used the LST as an indicator and SAR-based methods, alone or in combination with other measurements. Spectral information is, on the contrary, more susceptible to errors as waste disposal sites are often misclassified as urban areas or bare soil. Furthermore, several studies have shown that the dimensions of landfill sites are decisive for the analysis. As expected, small landfill areas are more difficult to identify, and this characteristic makes the detection of newborn waste disposal sites difficult. We can therefore assume that, generally speaking, RS-based methods are effective and efficient for the identification and monitoring of large and known waste disposal sites. On the contrary, the use of this technology is more challenging in the case of smaller sites. It follows that RS techniques perform less in tasks such as the early warning or detection of illegal dumps. Certainly, the use of very high-resolution images can overcome these limitations, but these are often not as widely and freely distributed as Landsat or Sentinel acquisitions.

## 2. Case Studies

Beginning from the review on the application of EO data for landfill monitoring, some relevant techniques were selected and tested on predetermined tests sites to demonstrate some application cases. The aim is to illustrate the behavior of the satellite-based measurements related to changes in the sites. In particular, the NDVI, NDWI, DDI and LST were selected for a multispectral data analysis, while for SAR data, the backscatter coefficient was considered. The analyses considered the above-mentioned indexes independently and in combination with each other.

As an approach, a multitemporal analysis was performed. Firstly, very-high-resolution (VHR) historical images from Google Earth were visually analyzed to define the sites’ geometries. Indeed, polygons were manually delineated through a visual analysis of these images. Moreover, the same images were inspected to observe possible changes in the sites over the past five years. This method allows for the comparison of images representing the same scene at different moments in time. Consequently, when changes affecting the site (e.g., land cover modification events and spatial variations within the sites’ perimeter) were visible, satellite data were downloaded and processed to observe the possible correlated variations of the multispectral indexes, LST, and SAR backscatter. As a result, to provide a complete overview of such variations, in this section it is possible to find panels representing the above-mentioned indexes as images centered on the study sites. In addition to this visual representation, to quantify what is observable from the images, numerical estimates based on the aforementioned indexes are provided. Particularly, the tables contain the mean values of indexes computed over the areas affected by change in the sites. These areas were delineated through a visual interpretation of the Google Earth VHR historical images.

Concerning multispectral data, Sentinel-2 acquisitions were involved. Notably, a single image per year was considered for the comparison so that all the acquisitions are related to the same year period to consider the seasonal effects on the surface reflectance. Regarding the LST, Landsat 8 images close to the Sentinel-2 acquisition dates assumed for the other optical indexes’ computations were considered and processed according to what was mentioned in Section 2.1.4. In addition, concerning the SAR data, Sentinel-1 images were considered to obtain the backscatter coefficient by downloading and processing the relevant products.

**Figure 2:**
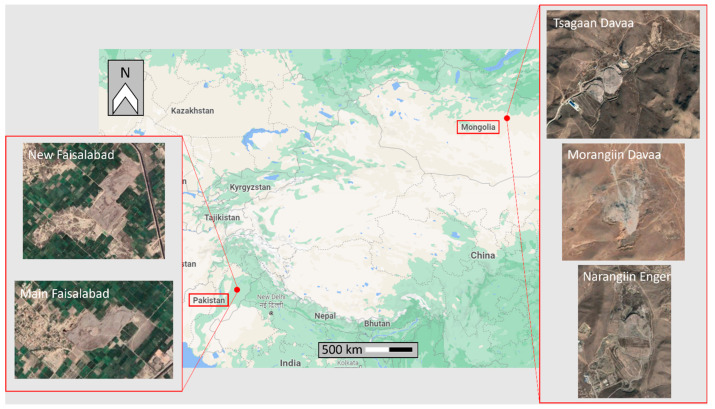
Location of the test sites. (**Left**): New Faisalabad and Main Faisalabad landfill sites located in Pakistan; (**Right**): Tsagaan Davaa, Morangiin Davaa, and Narangiin Enger landfill sites located in Mongolia.

Regarding the choice of the study sites, known dumping sites located in the regions of Pakistan and Mongolia were utilized as test sites. Indeed, as stated in the Introduction, MSW management represents a priority, especially in low- and middle-income countries, where the volume of waste generated is increasing rapidly, and engineered waste disposal systems are often not present. The regions of interest are reported in Figure 2, which shows the countries of Pakistan and Mongolia and the dumping site located in Pakistan, near the city of Faisalabad, and the three sites located in Mongolia, in the city of Ulaanbaatar.

Table 1 specified the products utilized for the analysis and the relative satellite acquisition platform. The area of acquisition is also reported.

### 2.1. Main Indexes and Parameters Used for the Analysis

#### 2.1.1. NDVI

The NDVI is the most known and used vegetation index for quantifying green vegetation by measuring plant health based on how a plant reflects light (usually sunlight) at specific frequencies. The NDVI calculation normalizes green leaf scattering in the near infrared (NIR) wavelength and chlorophyll absorption in the red wavelength (RED), according to the following expression:

(1) $$\mathrm{NDVI}=\frac{\mathrm{NIR}-\mathrm{RED}}{\mathrm{NIR}+\mathrm{RED}}$$

where NIR and RED are the reflectance provided by Sentinel-2 Level L2A band 8 and band 4 products, respectively [47].

The equation above always results in an NDVI value between −1 and +1. A number between −1 and 0 suggests an inanimate object, such as roads, buildings, water bodies, or dead plants. Values close to zero generally correspond to barren areas of rock, sand, or soil. Low, positive values represent unhealthy plants and stressed leaves (and scrubland or grassland as the type of vegetation). In contrast, high values indicate healthy plants and leaves (and dense evergreen forest as the type of vegetation).

#### 2.1.2. NDWI

The NDWI can also be considered an independent vegetation index complementary to the NDVI. The NDWI is sensitive to changes in the water content of vegetation canopies. The following equation (Equation (2)) describes how the NDWI is calculated:

(2) $$\mathrm{NDWI}=\frac{\mathrm{GREEN}-\mathrm{NIR}}{\mathrm{GREEN}+\mathrm{NIR}}$$

where GREEN and NIR are the reflectance provided by Sentinel-2 Level L2A band 3 and band 8 products, respectively [48].

The equation above results in an NDWI value between −1 and +1. A number between −1 and 0 suggests an inanimate object, such as roads, buildings, water bodies, or dead plants. Moreover, the higher the NDWI value between 0 and 1, the greater the vegetation canopies’ water content. Moreover, the NDWI is insensitive to atmospheric conditions.

#### 2.1.3. DDI and SAVI

The SAVI (soil-adjusted vegetation index) is a modification of the NDVI to take into account vegetation density [49], and it is defined as (Equation (3)):

(3) $$\mathrm{SAVI}=\frac{\left(\mathrm{NIR}-\mathrm{RED}\right)\times \left(1\cdot L\right)}{\mathrm{NIR}+\mathrm{RED}+L}$$

where NIR and RED are as defined above, and L is a canopy background adjustment factor, assumed equal to 0.5. The  SAVI is used to calculate the DDI. The latter is not a widely used index for RS applications since, in contrast to the indexes mentioned above, it is a specific indicator for dump detection and monitoring. Indeed, it extends the NDVI idea by using a soil-adjusted vegetation index and combining it with the local texture of an image at the waste site. The index is based on the following formula (Equation (4)):

(4) DDI=SAVI × Entropy

where Entropy is a statistical measure of randomness that can be used to characterize the texture of an input image around a target pixel [45]. In this context, it is applied to the vegetation index images through the gray-level co-occurrence matrix. (GLCM) [50].

#### 2.1.4. Land Surface Temperature

The LST is a key parameter that can be retrieved from TIR remotely sensed data. Many algorithms are available for LST estimation from satellite data. A method for surface temperature retrieval from Landsat data using a single band (Band 10) algorithm, based on the radiative transfer equation, has been implemented, e.g., in [51], in case of lakes, but it can be generalized to land surfaces by adopting appropriate values of surface emissivity. To this aim, the emissivity can be assumed on the reference of an empirical relation reported below (see Equation (5)), which was suggested in a previous work [52].

(5) ε=1.0094+0.047×ln(NDVI)

The proposed algorithm for the land surface temperature estimation consists of deriving the atmospheric corrected spectral radiance for a single Landsat thermal band, exploiting a NASA web tool which provides three parameters requested in the radiative-transfer-based Planck equation.

The first step in this workflow is radiometric calibration, which obtains the spectral radiances from the digital number values measured by sensors on board satellites (as shown in Equation (6)).

(6) $$L_{1}=\left(\frac{L_{\max\;\lambda }-L_{\min\;\lambda }}{{\mathrm{DN}}_{\max}-{\mathrm{DN}}_{\min}}\right)\times \left(\mathrm{DN}-{\mathrm{DN}}_{\min}\right)+L_{\min\;\lambda \;}\left[\frac{W}{m^{2}\cdot \mathrm{sr}\cdot µm}\right]$$

where L_1_ is the spectral radiance, DN is the digital number, L_min λ_ and L_max λ_ are the spectral radiances scaled on the base of DN_min_ (the minimum pixel value before radiometric calibration) and DN_max_ (the maximum pixel value before radiometric calibration) reported in the metadata of Landsat products.

Then, atmospherically corrected spectral radiance values can be obtained from the following Equation (7):

(7) $$L_{2}=\left(\frac{L_{1}-L↑}{\varepsilon \tau }\right)-\left(\frac{1-\varepsilon }{\varepsilon }\right)\times L{↓}_{\;}\left[\frac{W}{m^{2}\cdot \mathrm{sr}\cdot µm}\right]$$

where the atmospheric upwelling (L↑) and downwelling (L↓), radiance, and the total atmospheric transmission (τ) are derived from the NASA-developed web tool for correcting thermal bands in Landsat data, which can be accessed at the following link: http://atmcorr.gsfc.nasa.gov. (Last accessed on 24 February 2023).

The land surface temperature is finally obtained (see Equation (8)) by inverting Planck’s function, where K_1_ and K_2_ are constants reported in the metadata of each Landsat satellite product (e.g., K_1_ = 774.885 and K_2_ = 1321.079 for Landsat-8/TIRS).

(8) $$\mathrm{LST}=\frac{K_{2}}{\ln\left(\frac{K_{1}}{L_{2}}\right)}\left[K\right]\;$$

In order to mitigate possible year-dependent climatological effects, each LST map reported in the results was obtained by averaging the LST values retrieved through the aforementioned procedure applied to two Landsat products collected in a period consistent with the acquisition days of the images used for computing the other indexes considered for the analysis.

#### 2.1.5. Backscatter Coefficient

In literature, the SAR data employment revealed significant results, mainly by providing information about the structural changes occurring in landfills or, generally speaking, waste disposal sites. Indeed, since multispectral data provide details concerning the radiometric characterization of the observed surface, SAR acts as a complementary technology due to its geometry-based observables. Moreover, with SAR data, it is also possible to measure a variation occurring on the landfill surface when the optical signal is saturated or not exploitable because of weather conditions [45]. From an operational perspective, the radar signal is transmitted by the satellite down to the Earth’s surface and scattered back towards the satellite’s antenna by the Earth’s surface itself. Concerning this mechanism, SAR backscatter consists of part of that signal recorded by the onboard satellite’s sensor. Many factors related to the surface structure influence the SAR backscatter, including the surface’s roughness and its electrical conductivity (or dielectric constant). Rough surfaces will backscatter more radar energy back to the satellite radar antenna, while smooth surfaces will backscatter much less or even no energy back to the satellite radar antenna. A second influence on radar backscatter is due to the surface’s electrical conductivity (dielectric constant), which is directly related to the moisture content of the soil. A high moisture content means a high backscatter, and a low moisture content means a low backscatter. Another factor influencing the SAR backscatter is the local incidence angle, which is defined by the incident radar beam and the normal to the intercepting surface since reflectivity from distributed scatterers decreases with increasing local incidence angles. Concerning the backscatter analysis, a couple of SAR acquisitions related to the two years considered were processed to obtain the backscatter coefficient in both polarization (VH and VV). Regarding the processing, the two images were ingested in the SNAP software version 8 to progressively apply all the needed operators to obtain the backscatter coefficient. Particularly the following sequence of processing function were applied.

- Apply Orbit File;
- S-1 TOPS Split;
- Calibration;
- S-1 TOPS Deburst;
- Terrain Correction;
- Speckle Filter.

The above-mentioned functions represent the typical operators needed to process Sentinel-1 SLC data as described, e.g., in [53,54].

### 2.2. Single-Parameter-Based Analysis

#### 2.2.1. Vegetation-Indexes-Based Analysis

**Figure 3:**
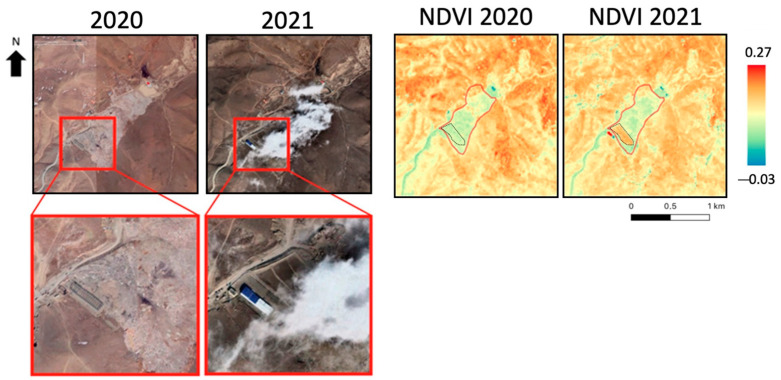
Tsagaan Davaa landfill site. (**Left**): Google Earth image over the area; (**Right**): NDVI map. Red line: landfill perimeter, black dashed line: area affected by the land cover change.

The Tsagaan Davaa landfill is located in Mongolia. A land cover change in the southernmost part of the landfill can be observed between 2020 and 2021, as shown on the left in Figure 3. Despite the cloud cover, the change was still visible. As can be found in the literature, the NDVI is a well-established index that is also frequently used in landfill detection because it can correlate vegetation stress to potential waste-induced contamination. For this purpose, the NDVI was applied to this site, and it is shown on the right side of the image before and after the event. The index clearly highlights the area of interest in both 2020 and 2021, reporting a lower value within the landfill than the surrounding areas. The event is also clearly represented by the NDVI index, which rose in 2021 when the change occurred. To quantify the increase in NDVI values, the mean values over the area where the land cover change occurred (black dashed polygons in Figure 3) were computed before and after the above-mentioned event, and the values are reported in Table 2. The results show an increase in the NDVI values, which was expected due to the removal of waste material and the resulting land cover change suggested by the visual interpretation of the Google Earth imagery.

**Figure 4:**
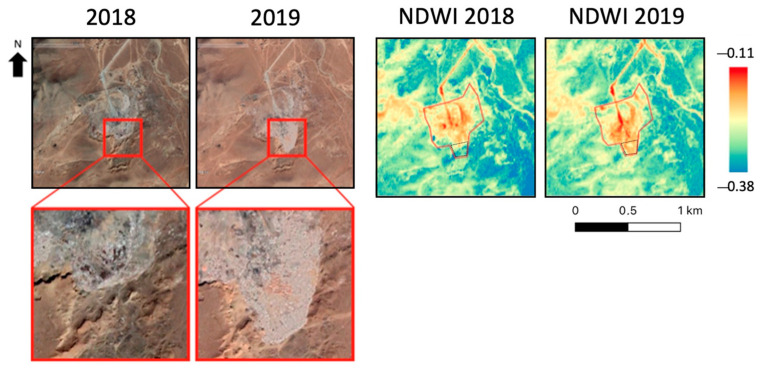
Morangiin Davaa landfill site. (**Left**): Google Earth acquisition over the area; (**Right**): NDWI map. Red line: landfill perimeter, black dashed line: area affected by the waste disposal.

The NDWI was analyzed for the Morangiin Davaa landfill in Mongolia, shown in Figure 4 on the left. The NDWI was used since the soil water content can be related to the leachate produced by waste. In this site, between 2018 and 2019, a waste disposal site expansion in the southern part occurred. An increase in the NDWI values is noted for the site’s southern part, according to expectations.

Moreover the above-mentioned NDWI increase can be observed not only in the illustration (Figure 4), but also by computing the mean values over the event-related area of the landfill (Table 3), suggesting the potential presence of leachates in the site, particularly in the area affected by the new waste disposal (in the southern area).

**Figure 5:**
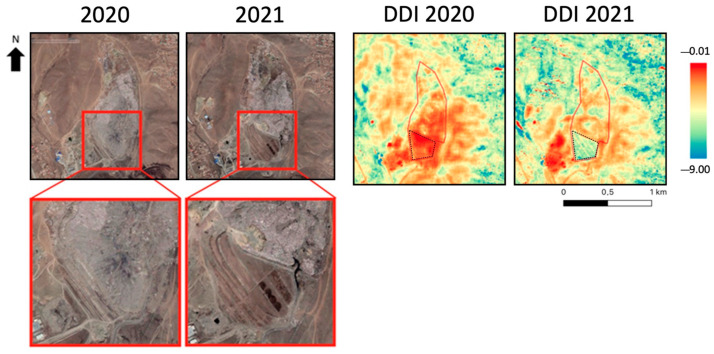
Narangiin Enger landifll site. (**Left**): Google Earth acquisition over the area; (**Right**): DDI map. Red line: landfill perimeter; black dashed line: area affected by the land cover change.

In the Narangiin Enger landfill, Mongolia, an event occurred between 2020 and 2021: a land cover change in the southern area of the site, shown on the left in Figure 5. The DDI was developed specifically for landfill detection, and an application example is shown for this site. The land cover change is distinctly detected since a decrease in DDI values in that specific area of the site was observed. Also in this case, the mean values over the southern area (black dashed polygon in Figure 5) of the site were computed before and after the land cover change (Table 4). A decrease from −1.12 to −5.65 occurred in the area of interest, confirming what was expected.

#### 2.2.2. Land-Surface-Temperature-Based Analysis

**Figure 6:**
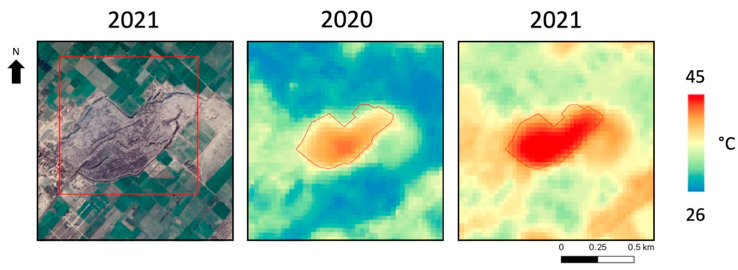
Main Faisalabad landfill site. (**Left**): Google Earth acquisition over the area; (**Middle**): LST map in 2020; (**Right**): LST map in 2021. Red line: landfill perimeter.

The LST was computed for the Main Faisalabad landfill in Pakistan (see Figure 6). It appeared that there were no evident changes in the surface temperature distribution in the images representing the two years considered and, overall, in the shape of the landfill, the LST was able to effectively delineate the perimeter of the landfill from the surrounding area. It can be noted that the LST values in 2021 were higher than those registered in the same period of 2020 for the same scene, but what is important to underline is that the spatial distribution of the LST values in the images as proportionally conserved. As previously mentioned, since there were no evident changes between 2020 and 2021, the mean values were computed over the entire site area to investigate the possibility of delineating the landfill perimeter using the temperature information. In this case, the comparison of the LST between the two years could be affected by differing annual climatology. To consider this effect, an overall temperature shift was computed between the two images by considering only the area outside the landfill, obtaining a shift value of 5.9 °C. As mentioned, it is worth focusing on the difference between the site’s temperature and the surrounding area’s temperature. For this purpose, the mean LST was computed inside (LST IN) and outside (LST OUT) the site to quantify the difference in both the two examined years. The results, shown in Table 5, suggest a temperature gap of about 5 °C that was conserved over time.

#### 2.2.3. SAR-Based Analysis

**Figure 7:**
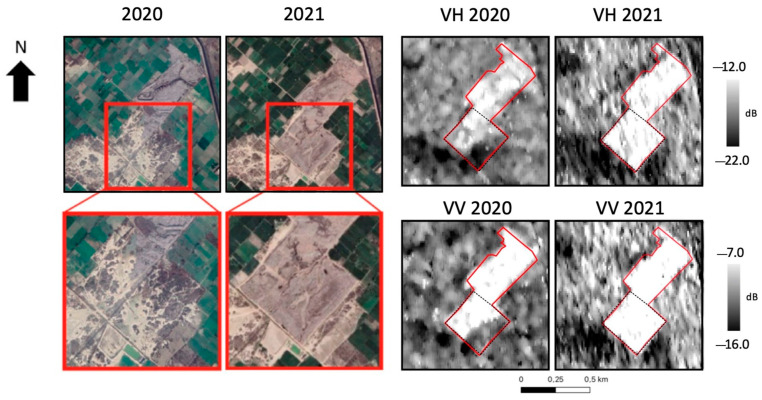
New Faisalabad landfill site. (**Left**): Google Earth acquisition over the area; (**Right**): backscatter maps in VH and VV polarizations. Red line: landfill perimeter.

Regarding the indexes derived from optical data, SAR images were processed to obtain the backscatter coefficient in VH and VV polarizations. In this regard, the site of New Faisalabad, located in Pakistan, was selected as a reference site for the analysis. The site is reported on the left side in Figure 7, as shown in Google Earth. In these images, a significant expansion in the southern area of the site can be observed between 2020 and 2021.

As shown in Figure 7, the backscatter highlighted the perimeter of the landfill, since high values of the backscatter coefficient occurred inside the site boundaries, while the surrounding area was characterized by lower values. The expansion of the site can be assumed, since the backscatter coefficient increased for both polarizations in the southern area of the site. The increasing backscatter coefficient can be attributed to a change in the scattering mechanism, confirming the expected surface variation suggested by the visual interpretation of the Google Earth images. Indeed, the surface of the area subject to the expansion changed from bare soil to a waste disposal that, assuming its heterogenous composition and geometry, brought an increase in the signal intensity measured by the radar sensor.

From the comparison represented in Table 6, it is possible to notice an increase of about 4 dB from 2020 to 2021 in both polarizations.

### 2.3. Multiple-Parameter-Based Analysis

SAR- and Multiple-Optical-Parameters-Based Analysis

Further to the above-illustrated analysis of the single selected parameters, i.e., multispectral indexes, the LST, and SAR-derived backscatter, their joint application is worth showing. In particular, for the sake of simplicity, the three vegetation indexes and the LST were computed on the site adopted for the SAR-based analysis, i.e., the New Faisalabad landfill.

**Figure 8:**
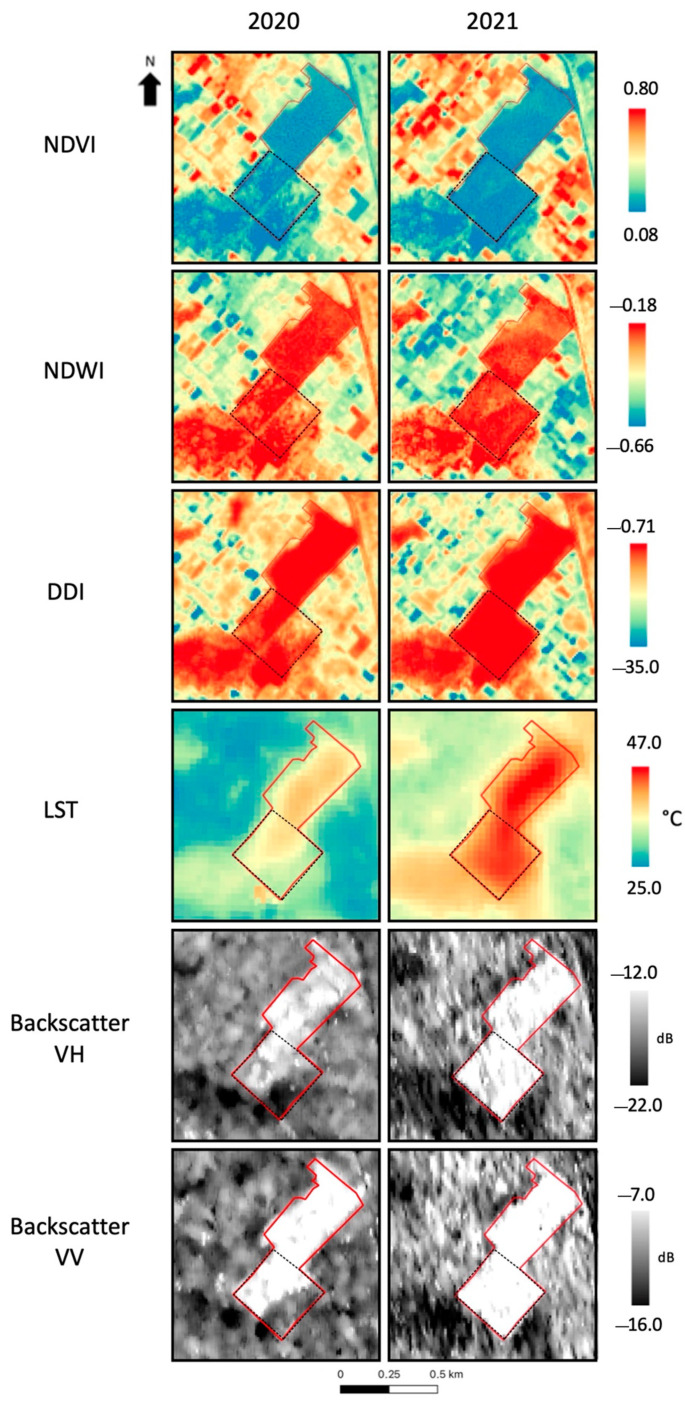
New Faisalabad landfill site. In sequence: NDVI, NDWI, DDI, LST and SAR backscatter in VH and VV polarizations. Red line: landfill perimeter; black dashed line: area affected by the waste disposal.

As represented in Figure 8, the three indexes, i.e., NDVI, NDWI, and DDI, revealed the dump area relative to its surroundings. However, the land cover pattern affected the accuracy of the results. For example, the large, bare soil surface outside the dump, in the bottom left corner of the images, reported values for the indexes comparable to those inside the dump. Despite this, the southern part of the area of interest showed a slight decrease in the NDVI and an increase in the NDWI and DDI in 2021, according to expectations regarding the change in the analysis. In addition, the pixel distribution of the three indexes appeared more homogeneous in the 2021 images, causing the dump boundaries to be more defined in the southern part due to the waste disposal that occurred.

As part of the analysis, the LST was computed from images acquired in the two considered years (see Figure 8). Despite the slightly different temperature ranges observed in the two years, it can be noted that the southern part of the landfill (which is the part under investigation) was affected by an increase in the LST values when compared to the surrounding areas. This outcome is aligned with the results obtained using the other indexes considered. 

Regarding the previous analysis, the mean values were computed over the area affected by the change (the southern area of the site, highlighted with a black dashed line in Figure 8) for 2020 and 2021. The computed values show an expected trend for all the indexes (Table 7). Particularly, the NDVI decreased while the NDWI increased by about 0.04 and 0.03, respectively. In addition, the DDI increased, showing a higher difference from 2020 to 2021 when compared to the other multispectral indices (3.78). As previously mentioned, the comparison between the two LST images acquired in different years is affected by a climatology-dependent shift. As in the previous case study, the shift was computed between the two images by considering only the area outside the landfill, obtaining a value of 6.2 °C. Moreover, to evaluate the temperature change due to the waste disposal, the LST was computed by averaging the images over the specific area, i.e., the southern part of the site. As a result, a difference of 8.5 °C was obtained so that if the climatology-dependent temperature shift is subtracted, an increase of 2.3 °C can be associated with the waste disposal that occurred. To provide an overview of the variations related to all the parameters, the SAR backscatter in both polarizations is also listed in Table 7.

### 2.4. Discussion

Due to the rapid urbanization process and the even more rapid increase in the volume of waste generated, efficient waste management is essential in current society. Globally, and especially in low- and middle-income countries, most of the waste produced is dumped or discarded in various types of landfills, which are not always equipped with appropriate systems to collect leachates and gases. These are often open dumps or uncontrolled landfills that pose a real threat to environmental protection and public health. The identification and monitoring of these types of waste disposal systems are required in order to improve MSW management. RS represents a valid method for landfill monitoring since it can overcome the difficulties caused by on site surveys, which are often time- and resource-consuming. Presently, RS platforms can carry a wide variety of heterogeneous sensors on board with different spatial and spectral resolutions, which are capable of distantly monitoring the Earth’s surface and extracting valuable information for this specific purpose.

This work reviewed the methods existing in the literature for the detection, mapping, and monitoring of landfill sites, both authorized and illegal, through satellite RS technologies. In fact, the large amount of available and often open-access data acquired by different satellite sensors has allowed for the development of these types of applications, making this topic a subject of increasing interest to the scientific community. An analysis of the literature showed that satellite RS data are sometimes used in combination with other methodologies, such as aerial photography, GIS support, and field surveying. Among the available satellite sensors, both passive, multispectral sensors and SAR sensors are considered. In the first case, a large number of the analyzed works made use of freely distributed Landsat data, but very high-resolution images, such as those acquired by the Pleiades and WorldView satellites, were also considered. Multispectral products are utilized to estimate well-known multispectral indexes, such as the NDVI and NDWI, or to derive specific indexes for dump detection, such as the DDI. Moreover, a wide number of studies make use of Landsat sensors to derive the LST. On the other hand, SAR sensors, including the instruments carried by Sentinel-1, Radarsat, Envisat, and Cosmo-SkyMed, are used to study variations occurring in the landfill sites. SAR images are mainly applied to derive information about water content and volume changes through SAR interferometry, or to monitor deformation by analyzing time-series acquisitions. The acquisition from optical and SAR sensors are often combined to exploit the complementary information or for visual inspection purposes. In addition, it must also be pointed out that other RS platforms, such as aircraft and lately, UAVs in particular, also exist and are used for a similar purpose and with similar equipment.

The results presented in the analyzed works demonstrate the validity of satellite RS-based methodologies. For example, thermal anomalies derived from the LST can aid in deriving landfill locations or waste disposals inside the landfills, and also in correlating the heat flux emitted from waste to the quantity of buried waste. The main drawback is that the LST can be affected by local temperature anomalies caused by precipitation or urban heat islands. Additionally, the vegetation health status obtained by the NDVI represents an aid in locating dumping areas and assessing their impact on the environment. The landfill spectral signatures can also be exploited for the generation of land cover maps, although they have been shown to be prone to misclassification with urban areas. Regarding SAR data, interferometry is used to generate elevation models to obtain the height of the waste volume. Furthermore, backscatter coherence is studied to correlate this parameter to changes in waste volume and the backscatter signal to obtain information about moisture content. These complementary parameters, derived from multispectral and SAR sensors, are often combined for a comprehensive analysis.

These state-of-the-art techniques were applied to selected landfill sites in order to provide an overall analysis. Hence, the NDVI, NDWI, and DDI were derived from Sentinel-2 images, the LST was obtained through Landsat sensors, and the backscatter coefficient taken from Sentinel-1 products. For the Tsagan Davaa site, the NDVI proved to be sensitive to the land cover change since the values significantly increased over the relevant area. The NDWI analyzed for Morangin Davaa also increased as expected in correspondence with the site spatial variation (a new waste disposal). It is worth noting that this index is strictly related to the presence of water or liquid material. This means that its sensitivity to landfill changes, as for the other considered indexes, can be influenced by the waste type and composition. Concerning the DDI, the analysis conducted for the site of Narangin Engeer showed a sensitivity of the index to land cover changes. Particularly, the DDI values drastically decreased when waste materials were not more visible in the VHR Google Earth image over the analyzed area. The LST, as demonstrated by the analysis of the Main Faisalabad landfill, also proved to be an effective measure for site identification. As mentioned in the related paragraph, the comparison of the LST values computed from images acquired in two different years was affected by a climatology-dependent shift, which must be considered. Taking into account this shift, using the LST images allowed for the separation of the landfill site from the surrounding area, proving that the LST can be useful for site identification and monitoring. Concerning the SAR-based analysis, which was conducted for the site of New Faisalabad, the backscatter coefficient was shown to be sensitive to the landfill structure since the delineation of the site seemed to have been properly distinguished from the surrounding area. Moreover, the occurrence of a new waste disposal appears to have been correlated to the increase in the backscatter (in the two polarizations, VH and VV) from both a visual and numerical perspective. Eventually, on the same site, a multiple-parameters-based analysis was performed. Particularly, the NDVI, NDWI, and DDI succeeded in highlighting the landfill area and the changes that occurred. Despite this, it is possible to note that nearby areas with particular land cover showed index values comparable to those inside the landfill. On the other hand, the LST and backscatter seemed to properly highlight the perimeter of the site and the variation occurring after the new waste disposal. 

In general, the measures obtained from the multispectral and SAR images can contribute to the identification of the landfill site and its changes. In particular, when compared to the optical indexes and LST products, SAR backscatter seems to be more sensitive to the geometry of the landfill sites since the radar signal strictly depends on possible changes of the scattering mechanisms occurring on the surface. Moreover, it is worth mentioning that the combined use of all the selected parameters can improve the quality and robustness of the analysis with respect to the landfill site evolution. Indeed, temporal comparisons obtained using only one of the parameters can be affected by some influencing factors. Particularly, as previously mentioned, the vegetation indexes may show similarities between the spectral response inside the landfill and some areas on the surrounding surface. On the other hand, the LST and SAR-derived backscatter can be affected by climatology-dependent temperature shifts and different soil conditions (e.g., soil moisture content), respectively. Given all these considerations, the multi-parameter approach may represent an enhanced monitoring strategy for landfill evolution. Specifically, if we consider the results obtained for the case study, a decrease in the NDVI values and an increase in all the other parameters could be associated with the occurrence of a new waste disposal.

## 3. Conclusions

Landfilling remains a common method used worldwide for waste disposal, despite causing several negative impacts to the environment. The leachate produced by the waste seeps into the ground surface, contaminating the water and soil, and the gases produced by the waste piles affect the air, impacting local settlements and the whole natural environment. It follows that the identification and monitoring of these sites is essential to ensure a high quality of life and to preserve the health of humans and all other species and their natural habitats.

This review focused on the use of sensors onboard satellite platforms for the remote identification and monitoring of dumping sites. The number of relevant studies in the state of the art demonstrates the growing interest of the scientific community in this important topic for environmental safety. The use of satellite sensors opens numerous possibilities, allowing for the exploitation of spectral-based indexes and measurements that can be retrieved from different kinds of multi- and hyperspectral sensors and SAR sensors. We hope that our work will help researchers attain a comprehensive overview of the potential of satellite data for landfill identification, which is also outlined by means of a short practical analysis. Moreover, future significant developments in monitoring landfills with remote sensors can be provided by the growing use of new platforms, i.e., UAVs, the information retrievable from recent satellite atmospheric missions, such as GhGSat and Sentinel-5P, and hyperspectral data, such as those provided by the PRISMA and EnMAP missions, which will lead to fruitful future advanced monitoring procedures in this field.

## Figures and Tables

**Table 1:** Identification of the considered products and related satellite platform.

Area	Product ID	Platform
Mongolia	S2B_MSIL1C_20180403T035539_N0206_R004_T48UXU_20180403T075550 S2B_MSIL2A_20190415T034539_N0211_R104_T48UXU_20190415T072335 S2B_MSIL2A_20200402T035539_N0214_R004_T48UXU_20200402T072357 S2B_MSIL2A_20210404T034529_N0300_R104_T48UXU_20210404T055850	Sentinel-2
Pakistan	S2B_MSIL1C_20200405T054639_N0209_R048_T43RCQ_20200405T092247 S2B_MSIL2A_20210331T054639_N0300_R048_T43RCQ_20210331T083756	Sentinel-2
	LC08_L2SP_150038_20200325_20200822_02_T1 LC08_L2SP_149038_20200403_20200822_02_T1 LC08_L2SP_150038_20210328_20210402_02_T1 LC08_L2SP_150038_20210413_20210423_02_T1	Landsat-8
	S1A_IW_SLC__1SDV_20200326T130416_20200326T130443_031847_03ACE3_78A8 S1A_IW_SLC__1SDV_20210414T130422_20210414T130450_037447_046A1C_37E5	Sentinel-1

**Table 2:** NDVI mean values computed over the southern area of the Tsagaan Davaa landfill site. An increase between 2020 and 2021 occurred due to the land cover change.

Index	2020	2021
NDVI	0.089	0.176

**Table 3:** NDWI mean values computed over the southern area of the Morangiin Davaa landfill site. An increase between 2018 and 2019 occurred due to the new waste disposal.

Index	2018	2019
NDWI	−0.304	−0.211

**Table 4:** NDWI mean values computed over the southern area of the Narangiin Enger landfill site. An increase between 2020 and 2021 occurred due to land cover change.

Index	2020	2021
DDI	−1.121	−5.645

**Table 5:** LST mean values computed inside the landfill site and in the surrounding area. The higher temperature inside the site compared to the surroundings was maintained over time.

Index	2020	2021
LST IN (°C)	38.319	42.943
LST OUT (°C)	29.475	35.382

**Table 6:** Mean values of the backscatter coefficient computed over the southern area of the New Faisalabad landfill site (where the waste disposal occurred).

Index	2020	2021
SAR VH (dB)	−16.380	−12.187
SAR VV (dB)	−9.033	−5.337

**Table 7:** Mean values of all the considered indices computed over the southern area of the landfill site (where the waste disposal occurred).

Index	2020	2021
NDVI	0.1634	0.121
NDWI	−0.232	−0.205
DDI	−5.196	−1.411
LST (°C)	34.641	43.186
SAR VH (dB)	−16.380	−12.187
SAR VV (dB)	−9.033	−5.337

## Data Availability

The data used for the analysis are free and open access data from the Copernicus Open Access Hub (https://scihub.copernicus.eu/ accessed on 31 August 2022) and United States Geological Survey (USGS) (https://earthexplorer.usgs.gov/ accessed on 12 February 2023).
